# Supplementary material for: Increased In Vivo Exposure of N-(4-Hydroxyphenyl) Retinamide (4-HPR) to Achieve Plasma Concentrations Effective against Dengue Virus
Source: Pharmaceutics. 2023 Jul 18;15(7):1974. doi: 10.3390/pharmaceutics15071974 (PMC10384639; doi:10.3390/pharmaceutics15071974)
Supplement: Supplementary file 1 [file pharmaceutics-15-01974-s001.zip › pharmaceutics-2476784-supplementary.pdf]

Martin, A.J. et al. "Increased *in vivo* exposure of N-(4-hydroxyphenyl) retinamide (4-HPR) to achieve plasma concentrations effective against dengue virus"

Supplementary Data

Table S1. Composition of formulations used in mouse PK studies.

Formulation*	Composition	Target Dose (mg/kg)	Dose Volume (mL/kg)
HC	92% w/v corn oil, 8% w/v polysorbate 80	20	3
MDE	5% v/v ethanol, 95% v/v RPMI 1640 containing 10% v/v FBS	20	8.8
HPMC-SV	0.5% w/v hydroxypropylmethyl cellulose, 0.4% v/v polysorbate 80, 0.5% v/v benzyl alcohol in water	20	3
N23	50% w/v Maisine CC, 10% w/v Ethanol, 40% w/v Tween 85	20	2
N25	25% w/v Maisine CC, 25% w/v Lauroglycol 90, 10% w/v Ethanol, 40% w/v Tween 85	20	3
IV	10% v/v DMSO, 90% v/v PEG400	2	2

*HC: human clinical; MDE: mouse dengue efficacy; HPMC-SV: hydroxypropylmethyl cellulose suspension vehicle; IV: intravenous

Table S2. Parameter estimates obtained by fitting a 2-compartment linear pharmacokinetic model to the 4-HPR IV data (- ABT from Figure 5).

Parameter	Estimate*
V1 (L/kg)	0.233
k10 (h^{-1})	1.20
k12 (h^{-1})	5.42
k21 (h^{-1})	0.561

*Estimates from 4-HPR IV data (see also Table 6).

Table S3. Statistical comparison of plasma AUC values. Analysis was based on the 95% z-confidence intervals for the dose-normalised $AUC_{0\text{-}30h}$ of 4-HPR in male C57Bl/6 mice following single-dose oral administration of the formulations presented in Figures 1, 3 and Supplementary Figure S1. Differences in exposure between formulations were deemed to be statistically significant where there was no overlap of the confidence intervals.

Formulation	95% z-Confidence Interval for Dose-normalised $AUC_{0\text{-}30h}$ (μM*h)
HC	8.77 – 10.7
HPMC-SV	11.7 – 13.3 [1]
MDE	28.0 – 38.2 [2]
(Non-dispersed) N23	31.6 – 39.6 [2]
(Non-dispersed) N25	40.5 – 45.4 [3]
(Pre-dispersed) N25	31.3 – 43.2 [2]

[1] Statistically different to HC. [2] Statistically different to HC and HPMC-SV
[3] Statistically different to HC, HPMC-SV, MDE and (non-dispersed) formulation N23

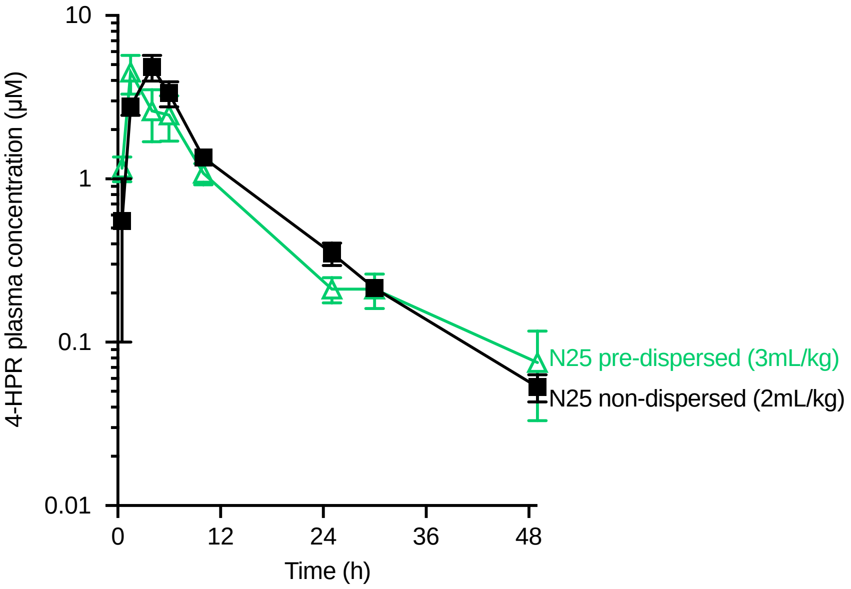

Figure S1. Pre-dispersion of lipid-based 4-HPR formulations does not enhance in vivo exposure. Mice were orally dosed with 20 mg/kg 4-HPR in self-emulsifying lipid-based formulation N25, either predispersed 1:2 (v/v) in water with vortex mixing; 3 mL/kg or non-dispersed (administered neat); 2 mL/kg. Data represent the mean ± SD of 3 mice per time point for the plasma concentration versus time. Parameter estimates are shown in Table S4.

Table S4. Summary of PK analysis for 4-HPR in male C57Bl/6 mice following single-dose oral administration using pre-dispersed and non-dispersed lipid formulations.

Parameter[1]	Lipid Formulation	
	Pre-dispersed N25[2]	Non-dispersed N25[2]
Average Dose (mg/kg)	21.2	21.8
Dose Volume (mL/kg)	3	2
Apparent $t_{1/2}$ (h)	10.5	8.4
Plasma $AUC_{0\text{-}inf}$ (h*μM)	36.2	46.3
Plasma C_{max} (μM)	4.5	4.8
Plasma T_{max} (h)	1.5	4.0
Bioavailability (%)	18	20

[1]Mice were dosed as per Figure S1. [2]Results are based on the mean of n = 3 mice per time point.
